# The use of duplex-specific nuclease in ribosome profiling and a user-friendly software package for Ribo-seq data analysis

**DOI:** 10.1261/rna.052548.115

**Published:** 2015-10

**Authors:** Betty Y. Chung, Thomas J. Hardcastle, Joshua D. Jones, Nerea Irigoyen, Andrew E. Firth, David C. Baulcombe, Ian Brierley

**Affiliations:** 1 Department of Plant Sciences, University of Cambridge, Cambridge CB2 3EA, United Kingdom; 2 Division of Virology, Department of Pathology, University of Cambridge, Cambridge CB2 1QP, United Kingdom

**Keywords:** ribosome profiling, duplex-specific nuclease, *Chlamydomonas*, mouse, translation

## Abstract

Ribosome profiling is a technique that permits genome-wide, quantitative analysis of translation and has found broad application in recent years. Here we describe a modified profiling protocol and software package designed to benefit more broadly the translation community in terms of simplicity and utility. The protocol, applicable to diverse organisms, including organelles, is based largely on previously published profiling methodologies, but uses duplex-specific nuclease (DSN) as a convenient, species-independent way to reduce rRNA contamination. We show that DSN-based depletion compares favorably with other commonly used rRNA depletion strategies and introduces little bias. The profiling protocol typically produces high levels of triplet periodicity, facilitating the detection of coding sequences, including upstream, downstream, and overlapping open reading frames (ORFs) and an alternative ribosome conformation evident during termination of protein synthesis. In addition, we provide a software package that presents a set of methods for parsing ribosomal profiling data from multiple samples, aligning reads to coding sequences, inferring alternative ORFs, and plotting average and transcript-specific aspects of the data. Methods are also provided for extracting the data in a form suitable for differential analysis of translation and translational efficiency.

## INTRODUCTION

Ribosome profiling measures at the codon level the extent to which individual mRNAs species of the transcriptome are engaged in protein synthesis. Initially developed by Ingolia et al. (2009), the method takes advantage of the knowledge that the position of a translating ribosome can be precisely determined by mapping the discrete, ∼30 nucleotide (nt) fragments protected by the ribosome from nuclease digestion (Wolin and Walter 1988). Ingolia et al. (2009) exploited advances in deep-sequencing technology to globally analyze ribosome-protected fragments (RPFs), generating high-resolution views of the location of translating ribosomes on the transcriptome at any one time (Ingolia 2010, 2014; Ingolia et al. 2009, 2011, 2012, 2013). Profiling has proven to be increasingly valuable in studies of the translation process, for example, in the discovery of novel open reading frames (ORFs), the determination of elongation rates, the identification of sites of ribosome pausing and in the study of protein folding (for review, see Morris 2009; Weiss and Atkins 2011; Michel and Baranov 2013; Ingolia 2014; Jackson and Standart 2015). It also has broad application in the analysis of global gene expression and has been exploited in studies of infectious diseases (Stern-Ginossar et al. 2012, 2015; Liu et al. 2013; Arias et al. 2014; Caro et al. 2014; Jensen et al. 2014; Muzzey et al. 2014; Vasquez et al. 2014; Yang et al. 2015), cell growth, differentiation and development (Brar et al. 2012; Huang et al. 2013; Lee et al. 2013; Stadler and Fire, 2013; Stumpf et al. 2013; Subramaniam et al. 2013; Baudin-Baillieu et al. 2014; Brubaker et al. 2014; Duncan and Mata 2014; Gonzalez et al. 2014; Hendriks et al. 2014; Katz et al. 2014; Kronja et al. 2014; Schrader et al. 2014; Vaidyanathan et al. 2014; de Klerk et al. 2015), apoptosis (Wiita et al. 2013), mitochondrial gene expression and disease (Rooijers et al. 2013; Williams et al. 2014), cell stress (Gerashenko et al. 2012; Labunskyy et al. 2014; Zid and O'Shea 2014; Sidrauski et al. 2015), cell toxicity (Haft et al. 2014), and cell evolution (Artieri and Fraser 2014; McManus et al. 2014).

**FIGURE 1.**
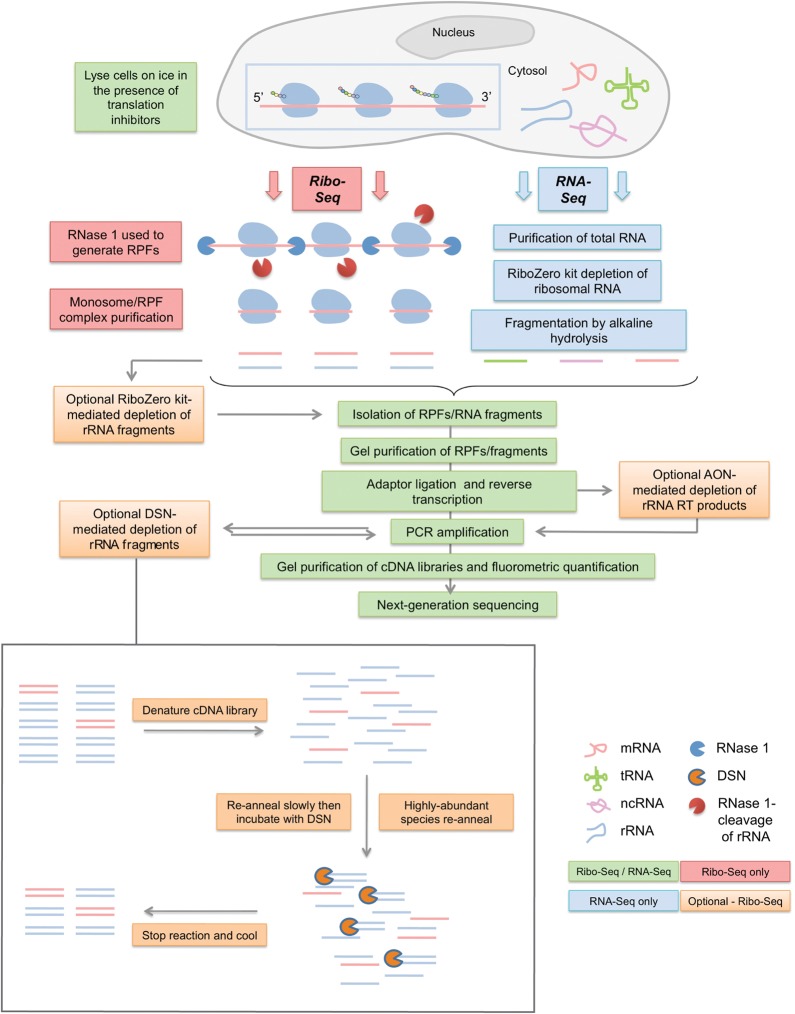
Ribosomal profiling strategy and points of rRNA depletion. The profiling methodology was based largely on that described by Ingolia and colleagues (Ingolia et al. 2009, 2012) except, following purification of ribosome-protected fragments (RPFs), library amplicons were constructed using a small RNA cloning strategy (Guo et al. 2010). Steps in the protocol specific to Ribo-seq, RNA-seq, or present in both are color-coded as indicated. Tested rRNA removal strategies are shown in orange boxes, with DSN treatment detailed separately at the *bottom* of the figure.

In the ribosome profiling methodology (Fig. 1), often referred to as Ribo-seq, cells are lysed under conditions optimized to minimize further ribosome movement (addition of translation inhibitors, rapid freezing), the lysate is treated with ribonuclease (often RNase 1) to degrade regions of mRNAs that are not physically protected, and the ribosomes harvested on sucrose gradients or through a sucrose cushion. The ribosome pellet is de-proteinized, the RPFs harvested by elution from a polyacrylamide gel, ligated to adapters, subjected to RT-PCR, deep sequenced and mapped back to the genome to reveal the location and abundance of ribosomes on mRNAs. The transcriptome itself is determined from the same lysate; total RNA is harvested, fragmented, cloned, and sequenced to generate an RNA-seq library.

Despite its increasing use, Ribo-seq is still in development (e.g., see Gerashcenko and Gladyshev et al. 2014; Gao et al. 2015) and there remain issues which limit its application. Not least among these is the problem of rRNA contamination, which can account for >90% of total reads (Gerashchenko et al. 2012). Hybridization-subtraction methods have been developed to reduce levels of major rRNA contaminants, but these are only partially effective, are time consuming, and can potentially introduce bias (Ingolia et al. 2011; Gerashchenko et al. 2012; Wickersheim and Blumenstiel 2013; Bazzini et al. 2014; Subtelny et al. 2014). Treatment of monosomes with EDTA, which dissociates the subunits, as well as minimizing the size range of RPFs sliced from polyacrylamide gels can also reduce rRNA contamination (Guo et al. 2010), but at the risk of losing important information that can be derived from analysis of a broader RPF size range (Rooijers et al. 2013; Lareau et al. 2014). Here we describe the use of duplex-specific nuclease (DSN) as a simple, species-independent way to achieve significant reductions in rRNA contamination. DSN, isolated from the hepatopancreas of the Kamchatka crab (*Paralithodes camtschaticus*), cleaves double-stranded DNA and RNA–DNA hybrid duplexes, with increased activity on perfectly matched duplexes (Shagin et al. 2002). DSN has been used in the normalization of cDNA libraries prior to next generation sequencing (Shagina et al. 2010) and the depletion of rRNA from RNA-seq libraries (Christodoulou et al. 2011; Yi et al. 2011; Matvienko et al. 2013; Miller et al. 2013). These experiments exploited the knowledge that the rate of DNA hybridization is proportional to the product of the concentration of the two separate DNA strands. Following denaturation of an RNA-seq cDNA (amplicon) library, the most abundant sequences re-anneal first and can be selectively degraded by addition of DSN (at 68°C), while less abundant sequences remain as ssDNA (Yi et al. 2011). To test the effectiveness of DSN in ribosome profiling, we generated Ribo-seq libraries from mouse tissue culture cells and from the green alga *Chlamydomonas reinhardtii*. We found that DSN reduced rRNA contamination substantially with only slight depletion of the most abundant mRNA RPF species, even within libraries of *C. reinhardtii*, whose transcriptome is highly GC-rich (Merchant et al. 2007).

Another limitation of Ribo-seq is in data analysis, which requires considerable expertise in bioinformatics. Programs that allow non-specialists to easily interpret Ribo-seq data sets have only recently become available (Crappé et al. 2015; Legendre et al. 2015) and many analyses are not yet supported in published packages. Here we describe an R package riboSeqR (released under Bioconductor, 2014) that provides a set of methods for parsing ribosomal profiling data from multiple samples, aligning to coding sequences, inferring alternative reading frames, and plotting average and transcript-specific behavior of these data. A unique feature of RPFs when aligned to the transcriptome is that they reflect the triplet periodicity of the translation process, where, during elongation, the ribosome moves in steps of three nucleotides (i.e., one codon) at a time along the mRNA. By analyzing the phase of the triplet periodicity of aligned RPFs, it is possible to determine the reading frame of translation on an mRNA. This is particularly relevant for identifying short and/or non-AUG initiated ORFs and for characterization of translated ORFs which may overlap the “main” coding ORF, or be present downstream (Michel et al. 2012; Dunn et al. 2013; O'Connor et al. 2013). Thus, riboSeqR uses this feature to identify unannotated coding ORFs.

We tested and validated the package using data derived from the libraries described above. In addition to processing and displaying profiling data, the riboSeqR package also allowed us to visualize a variety of translational control events. The use of DSN and the riboSeqR package facilitates the application of ribosome profiling and will be of value to both old and new users of the technique.

## RESULTS

### Duplex-specific nuclease: a sequence-independent rRNA depletion strategy

**FIGURE 2.**
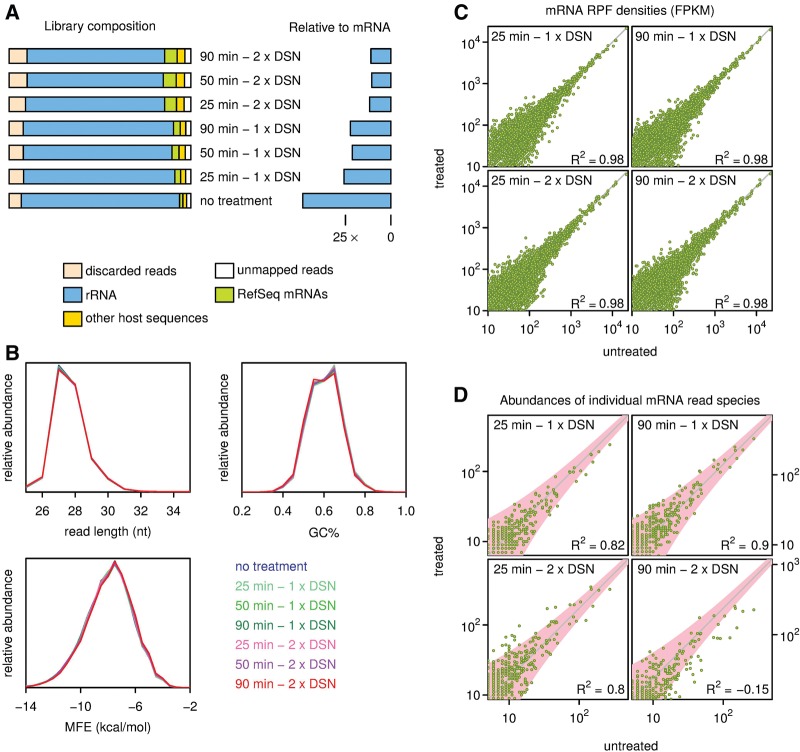
Analysis of the DSN-based rRNA depletion strategy using *Chlamydomonas* samples. (*A*) Relative rRNA depletion for different DSN treatments. Library composition is shown on the *left* and the amount of rRNA contamination relative to mRNA is on the *right* (see also Table 1). (*B*) Read abundance in DSN-treated and untreated libraries expressed as a function of read length, GC composition, and minimum free folding energy (MFE) for reads mapping to mRNAs. (*C*) RPF densities based on all RPFs mapping to NCBI RefSeq mRNAs for DSN-treated and untreated samples, expressed as fragments per kilobase per million mapped reads (FPKM). (*D*) Abundances of distinct mRNA-derived read species in DSN-treated and untreated samples. The gray guideline indicates the expected relationship if there is no depletion of mRNA—the slope is the ratio of the total number of mapped mRNA-derived reads in each sample. A theoretical 95% envelope based on χ^2^ statistics is shown in pink. *R*^2^ is calculated for distinct RPF species that have >5 occurrences in the untreated sample and is relative to the expected relationship indicated by the gray line (not a linear regression line), hence the potential for negative *R*^2^ values.

Ribo-seq and RNA-seq libraries were prepared from mouse tissue culture cells and the green alga *Chlamydomonas reinhardtii* and sequenced on MiSeq or HiSeq 2000 platforms (Table 1). The protocol, detailed in Figure 1 and Materials and Methods, includes a smallRNA cloning step to allow inexpensive in-house adapter activation and is adapted to Illumina smallRNA v2 to facilitate multiplexing. DSN treatment was performed at the library amplicon stage (post-RT-PCR; see Fig. 1). For each library, either one or two cycles of denaturing, annealing, and DSN treatment were performed; for *Chlamydomonas*, each treatment was carried out for 25, 50, or 90 min; for the mouse library, the reaction was for 25 min only. As shown in Table 1 and Figure 2A, for *Chlamydomonas,* each treatment decreased the proportion of rRNA substantially, increasing the proportion of mRNA in the sample by about fourfold after the two treatments. We did not see a noticeable effect of the time of incubation on the amount of rRNA depletion (Fig. 2A). In considering the use of DSN for depletion of rRNA from these libraries, we were mindful that post-hybridization nuclease treatment could potentially introduce biases arising from digestion of abundant mRNA-derived cDNAs. The possibility that DSN could also deplete annealed, GC-rich cDNAs, or single-stranded cDNA with a high propensity to form intramolecular structures was also considered. The highly GC-rich *Chlamydomonas* transcriptome (Merchant et al. 2007; Fig. 2B) was especially relevant in this regard, although the preferential activity of DSN on perfectly matched duplexes (Shagin et al. 2002) limited our concerns somewhat. An analysis of the physical profile of the reads is presented in Figure 2B, showing the length, GC content, and minimum free folding energy distributions of mRNA-derived RPFs for DSN-treated and untreated samples. The profiles of the different samples were found to be almost identical, indicating that DSN treatment introduces negligible bias with respect to these parameters for mRNA-derived RPFs. RPF densities on mRNA transcripts were found to closely follow a zero-intercept linear relationship when DSN-treated samples were compared with untreated samples, indicating that DSN did not noticeably deplete the most abundant mRNA transcripts (*R*^2^ = 0.98; Fig. 2C). We also compared the abundances of individual mRNA read species between untreated and DSN-treated samples (Fig. 2D). Due to the lower counts involved, *R*^2^ values were lower. However, there was relatively little depletion of abundant read species except for the 2 × 90 min treated sample.

**TABLE 1.**
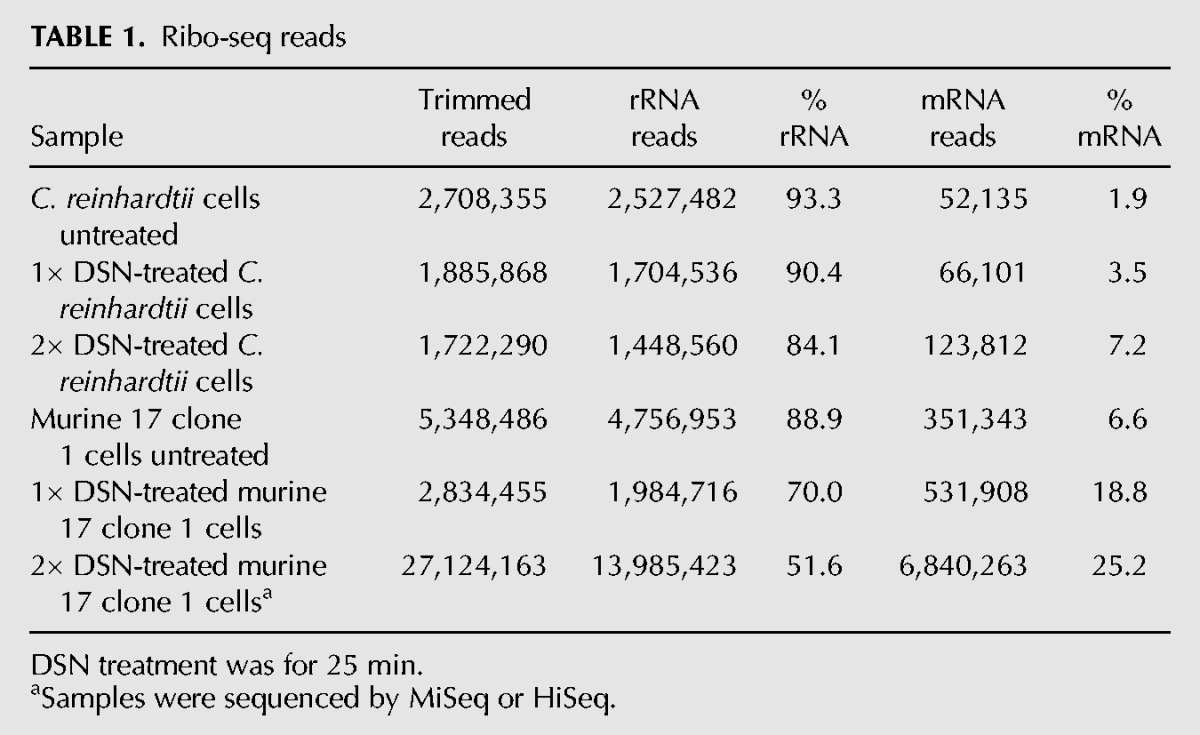
Ribo-seq reads

**FIGURE 3.**
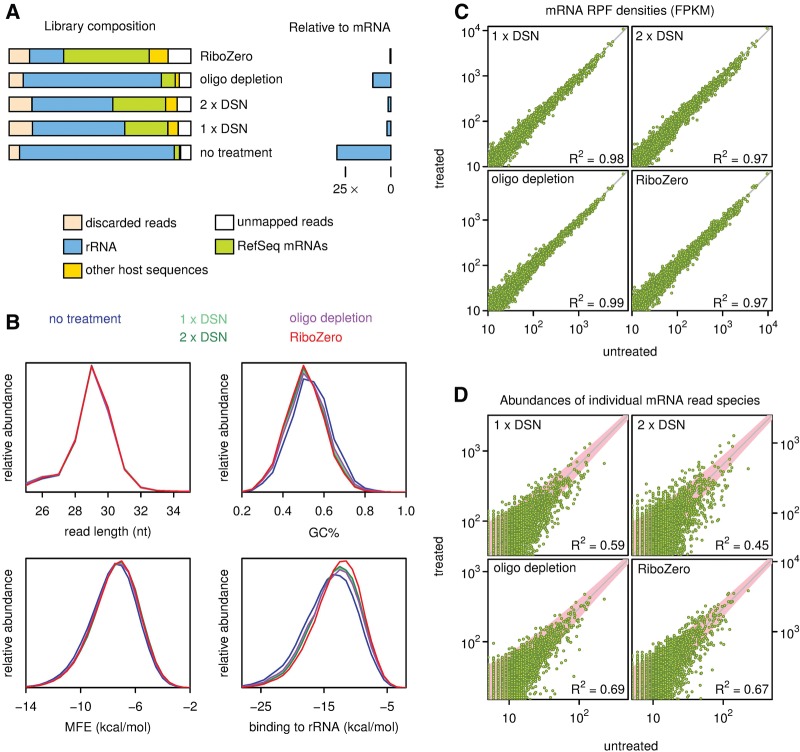
Comparison of rRNA depletion strategies using mouse samples. (*A*) Relative rRNA depletion for different depletion strategies. Library composition is shown on the *left* and the amount of rRNA contamination relative to mRNA on the *right* (see also Table 2). (*B*) Read abundance in treated and untreated libraries expressed as a function of read length, GC composition, minimum free folding energy (MFE), and optimal binding energy to reverse-complemented rRNA (essentially the RiboZero probe) for reads mapping to mRNAs. (*C*) RPF densities based on all RPFs mapping to NCBI RefSeq mRNAs for treated and untreated samples, expressed as fragments per kilobase per million mapped reads (FPKM). (*D*) Abundances of distinct mRNA-derived read species in treated and untreated samples. The gray guideline indicates the expected relationship if there is no depletion of mRNA—the slope is the ratio of the total number of mapped mRNA-derived reads in each sample. A theoretical 95% envelope based on χ^2^ statistics is shown in pink. *R*^2^ is calculated for distinct RPF species that have >5 occurrences in the untreated sample, and is relative to the expected relationship indicated by the gray line.

To directly compare the efficacy of DSN with other rRNA depletion strategies, we prepared four new mouse libraries, two that had been subjected either to one or two cycles of DSN (25 min), and a further two in which rRNA removal was achieved using either a pool of antisense oligonucleotides (AON) (Ingolia et al. 2012) or a RiboZero kit. The step in library preparation where these depletion methods were used is indicated in Figure 1. Libraries were sequenced on the NextSeq platform and RiboSeq read counts are displayed in Table 2. Treatment with RiboZero produced the greatest increase in library mRNA fraction (18-fold), followed by 2× and 1× DSN (11- and ninefold, respectively), while AON depletion gave only a threefold increase in mRNA fraction (Fig. 3A; Table 2). Again, DSN did not introduce any bias relative to mRNA read length or minimum free folding energy (Fig. 3B), although for unknown reasons in this experiment, all treatments led to a very slight bias in GC% compared with the untreated sample (Fig. 3B). As before, DSN did not noticeably deplete mean RPF densities for the most abundant mRNAs (*R*^2^ = 0.97–0.98; Fig. 3C). When we compared the abundances of individual mRNA read species, DSN was found to introduce more variability than RiboZero (*R*^2^ = 0.67 for RiboZero; 0.59 and 0.45 for 1× and 2× DSN, respectively; Fig. 3D). Individual RPF species showing evidence of depletion (below the diagonal line in Fig. 3D; 1 × DSN panel) tended to have slightly higher GC content (56.2% GC) than those without depletion (above the diagonal in Fig. 3D; 1 × DSN panel) (51.7% GC). Thus, DSN depletion may lead to slight underestimates of ribosome density at a few specific sites (e.g., strong ribosome pause sites in highly expressed transcripts); however, for the vast majority of applications, this is unlikely to be problematic. RiboZero on the other hand was found to introduce more bias than DSN for mRNA-derived reads that have stronger binding potential to the RiboZero probe (Fig. 3B, lower right panel). Given the robustness and specificity of rRNA depletion by DSN in the context of Riboseq, we anticipate this sequence-independent approach will allow application of ribosome profiling to a wide array of organisms.

**TABLE 2.**
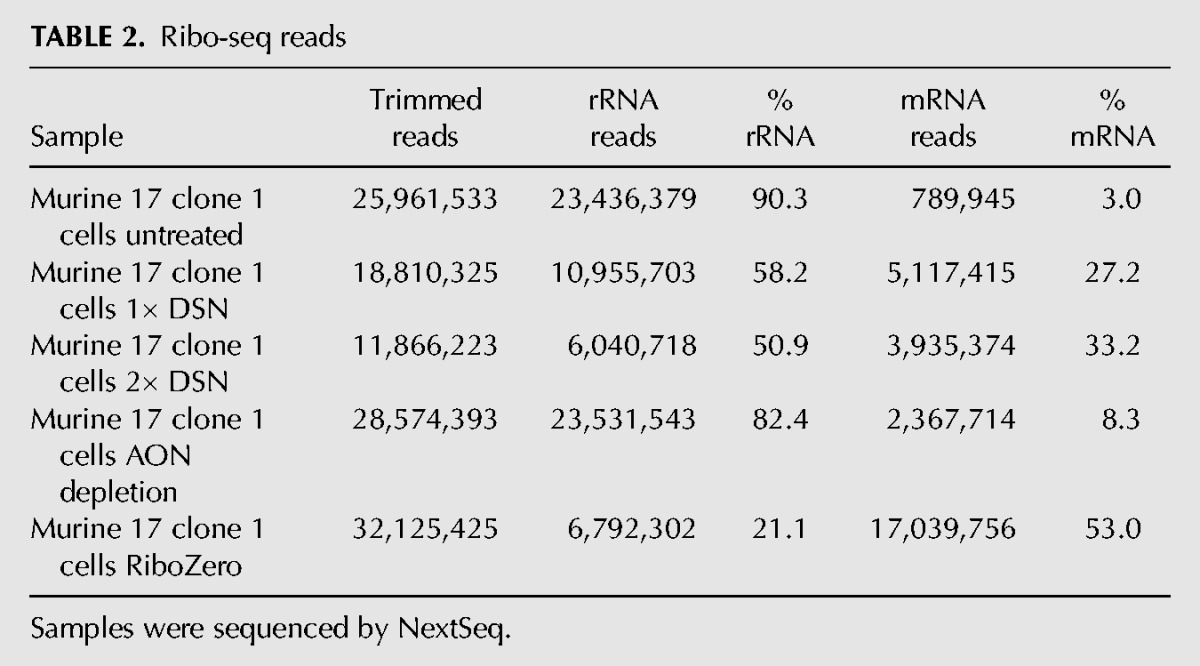
Ribo-seq reads

### A user-friendly bioinformatic package for Ribo-seq processing; riboSeqR

We have developed the riboSeqR R package (available at the Bioconductor website: http://www.bioconductor.org/packages/riboSeqR and also implemented at http://ribogalaxy.ucc.ie/) to provide a set of methods for user-friendly analysis of ribosome profiling data. The package parses data aligned to a (potentially de novo) transcriptome, providing frame-calling and plotting functions. The package optionally identifies potential coding sequences based on the identification of start/stop codons within the sequence of FASTA files, with RPFs mapping in-frame to corresponding ORFs. Alternatively, known coding sequences can be used. The versatility of this package is illustrated here through analyses of the *Chlamydomonas* and mouse Ribo-seq data sets, with a combined size of RNA-seq and Ribo-seq alignment files, respectively, of 1.2 GB and 2.3 GB. Scripts used to perform these analyses are provided in Supplemental Figures S1 and S2, and the run-time for these analyses was ∼14 min for *Chlamydomonas* and 31 min for mouse data, on a single 2.50 GHz processor with 16 GB of RAM.

**FIGURE 4.**
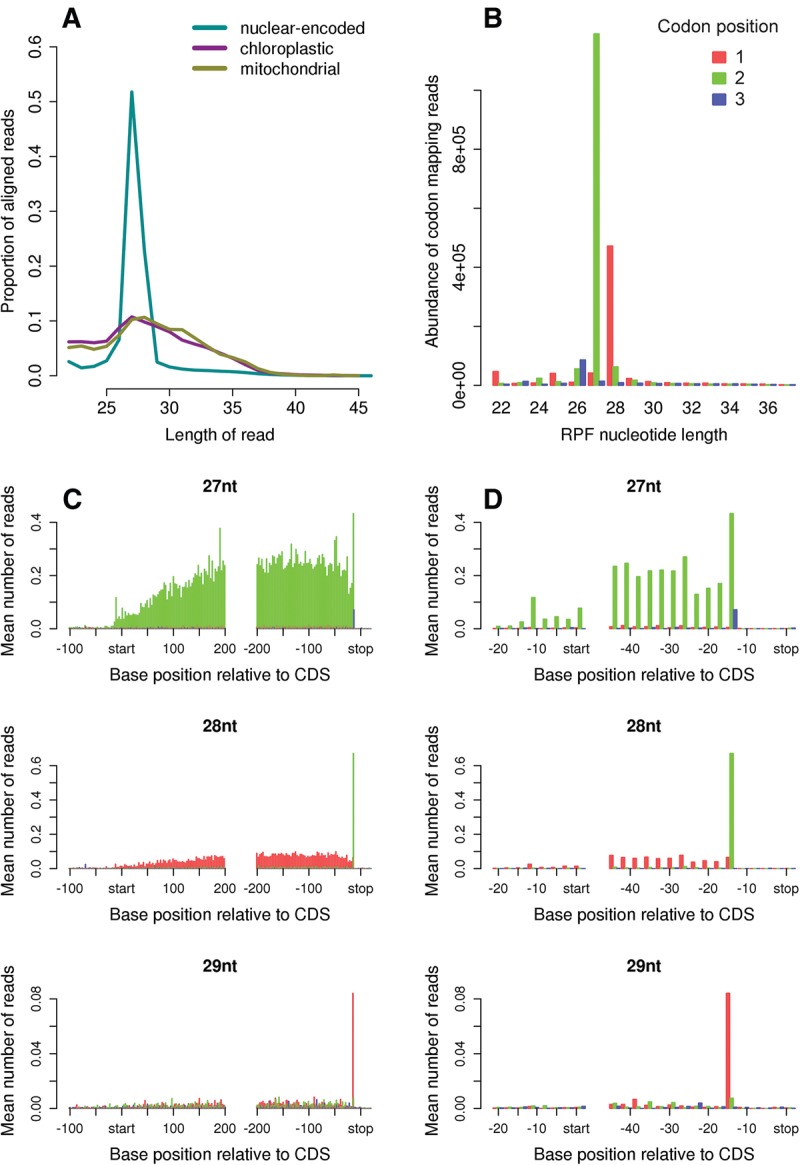
Ribosome profiling of *C. reinhardtii*. (*A*) Length distributions of RPFs mapping to the interior regions of nuclear-encoded, mitochondrial and chloroplastic coding ORFs. (*B*) Histogram of the codon positions (i.e., first [red], second [green] or third [blue] nucleotide of each N_1_N_2_N_3_ codon) to which the 5′ ends of RPFs map, as a function of RPF size class, for RPFs mapping to the interior regions of nuclear-encoded coding ORFs. (*C*) Histograms of RPF 5′ end positions relative to start and stop codons, for 27-, 28-, and 29-nt RPFs mapping to nuclear-encoded mRNAs. Coloring indicates the codon positions of the 5′ ends of RPFs. (*D*) Enlarged view around the start and stop codons; “start” and “stop” indicates the first nucleotide of the start and stop codon positions.

**FIGURE 5.**
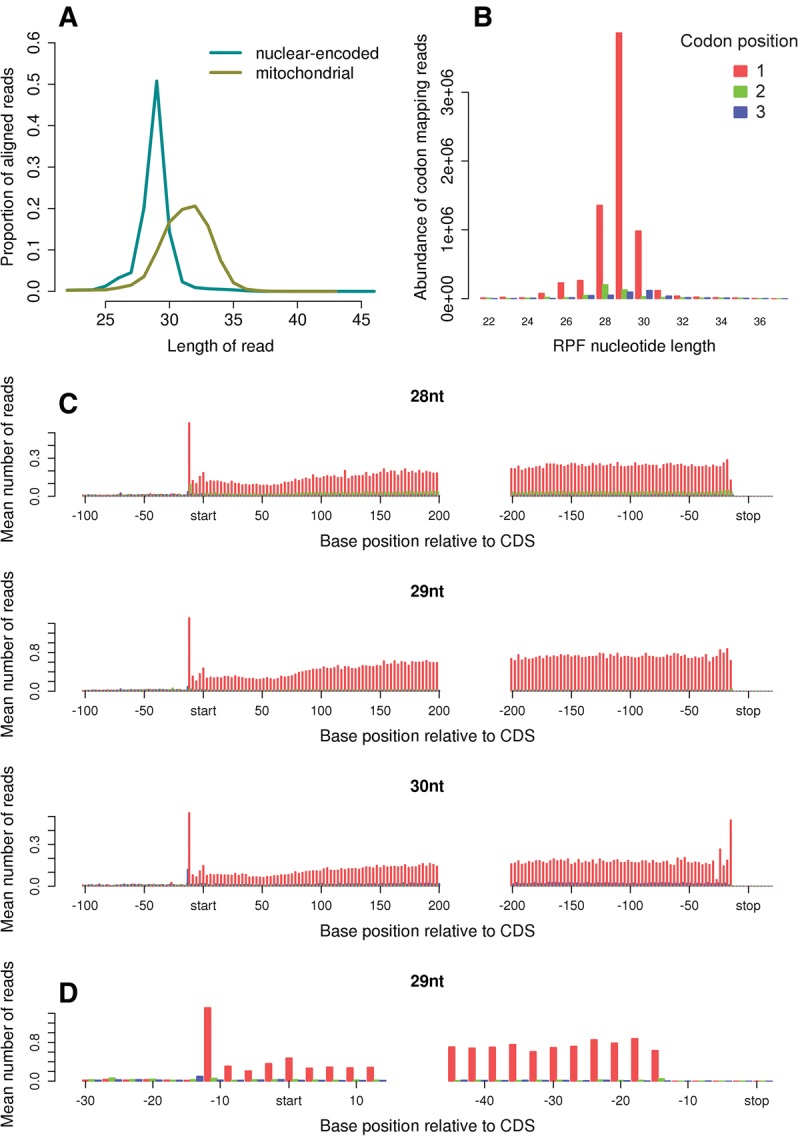
Ribosome profiling of mouse cells. (*A*) Length distributions of RPFs mapping to the interior regions of nuclear-encoded and mitochondrial coding ORFs. (*B*) Histogram of the codon positions to which the 5′ ends of RPFs map as a function of RPF length size class, as in Figure 4B. (*C*) Histograms of RPF 5′ end positions relative to start and stop codons, for 28-, 29-, and 30-nt RPFs mapping to nuclear-encoded mRNAs. Coloring indicates the codon positions of the 5′ ends of RPFs. (*D*) Enlarged view around the start and stop codons for the 29-nt size class. Deviations from the mean for peaks located 3–4 codons upstream (downstream) of the termination (initiation) site likely result from ligation and nuclease biases: RPFs whose 5′ ends align to these positions have constant nucleotides at or near to the 3′ (5′) end because of the conserved stop (start) codon; whereas, at other positions in the histogram, any ligation or nuclease biases deriving from the identity of the nucleotides at the termini of the RPFs are averaged out when averaging over different mRNA species.

We began the riboSeqR analysis by examining read-length distributions. For *C. reinhardtii*, which possesses chloroplastic, mitochondrial, and cytoplasmic ribosomes similar to plant ribosomes (Manuell et al. 2007), RNase 1 treatment typically produced cytoplasmic RPFs with a length size distribution sharply peaked at 27–28 nt (Fig. 4A). For the 27-nt size class, the 5′ ends of *C. reinhardtii* RPFs mapped overwhelmingly to the second nucleotide position of codons (Fig. 4B). For the second most abundant RPF size class, i.e., 28 nt, a large majority of 5′ ends mapped to the first nucleotide of codons, indicating, in this case, the addition of one nucleotide at the 5′ end of such RPFs relative to 27-nt RPFs (Fig. 4B). riboSeqR uses this initial analysis to filter the data, considering for further analysis those RefSeq annotated coding sequences that contain at least fifty 27-nt reads, mapping to at least 10 distinct locations within the coding region. Note that for highly translated coding regions, the small proportion of out-of-phase reads may cause overlapping but out-of-phase putative coding regions to pass this filtering step. riboSeqR thus further filters those putative coding regions by identifying those cases where the phase with the maximum number of reads (the maximal phase) is not the expected phase for that putative coding region. If the ratio of reads in the expected phase to maximal phase does not significantly (χ^2^ test with significance threshold of 0.05) exceed that ratio observed for all coding regions (Figs. 4B, 5B), the putative coding region is excluded from further analysis.

Using these selected coding sequences, riboSeqR constructs the weighted average number of n-nucleotide reads around the annotated coding start and stop sites (Fig. 4C). Contributions of reads from individual coding sequences are down-weighted by the total number of n-nucleotide reads per coding sequence length to avoid highly translated coding regions unduly influencing the profile. These plots further demonstrate the high level of triplet periodicity as a function of read size class. More importantly, they allow more detailed observations to be made concerning the behavior of the ribosome, especially at the sites of translation initiation and termination. During termination, the incorporation of release factors into the ribosomal pretermination complex induces a structural rearrangement that results in a footprint ∼1–2 nt larger relative to the footprint of the initiating or elongating ribosome (Wolin and Walter 1988; Alkalaeva et al. 2006). This change is clearly apparent in the riboSeqR figures generated from our data sets. In Figure 4C, the majority of RPFs in interior regions of coding sequences (i.e., elongation-state RPFs) have a length of 27 nt, with the modest read peak corresponding to terminating ribosomes most likely reflecting ribosomes paused at the stop codon with an unoccupied A-site. Based on the positions of the maximum values near to the start and stop codons, we can infer that, for 27-nt RPFs, the ribosome protects 11 nt 5′ of the P-site codon and 10 nt 3′ of the A-site codon (e.g., Fig. 4D). Pausing during termination leads to a much greater density of 28-nt RPFs at stop codons compared with interior positions (Fig. 4C). The 28-nt RPFs that map to stop codons most likely derive from ribosomes that have bound release factor complex. Again, relative to the density in interior positions, a still higher termination peak is apparent for the 29-nt RPFs size class (Fig. 4C; enlarged view in Fig. 4D). Based on the position at which the 5′ ends of RPFs map, it is apparent that the 28-nt termination RPFs involve a single nucleotide addition at the 3′ end, the 28-nt elongation RPFs involve a single nucleotide addition at the 5′ end, and the 29-nt termination RPFs involve one nucleotide addition at each end, all relative to 27-nt elongation-state RPFs (Fig. 4C,D).

We next used the same riboSeqR processing steps to analyze data from mouse cells (Fig. 5). Murine cytoplasmic RPFs had a length distribution typically peaking at 28–30 nt (Fig. 5A), ∼2 nt longer than the *C. reinhardtii* cytoplasmic RPFs. There is a precedent for such a difference because the wheat germ ribosome has an mRNA footprint some 2–4 nt smaller than the rabbit reticulocyte ribosome (Wolin and Walter 1988). The length distribution of mouse mitochondrial RPFs was broader than that of cytoplasmic RPFs, and shifted to longer length classes (Fig. 5A). We did not observe the bimodal peak for mitochondrial RPFs seen in a previous study (Rooijers et al. 2013), but this could be a consequence of size selection at the gel-purification stage that was not tailored specifically for organelle RPFs. For the murine sample, the 5′ ends of 87.4% of all RPFs mapping to interior regions of annotated coding sequences of nuclear-encoded mRNAs mapped to the first nucleotide of codons (Fig. 5B,C). For the most abundant length size class (29 nt), 94.0% of RPF 5′ ends mapped to this codon position. Such RPFs contain 12 nt 5′ of the P-site codon and 11 nt 3′ of the A-site codon (Fig. 5D). At termination codons, there was a noticeable decrease in the number of 28-nt RPFs and a substantial increase in the number of 30-nt RPFs (Fig. 5C), again illustrating the larger footprint of terminating ribosomes relative to elongating ribosomes. Similarly to *C. reinhardtii*, this increased RPF size corresponded to addition of a nucleotide at the 3′ end of RPFs (Fig. 5C).

### De novo inference of coding regions

Ribosome profiling allows the de novo annotation of coding regions within a transcriptome. We used riboSeqR to identify regions of the transcriptome beginning and ending with canonical start (AUG) and stop (UAG, UAA, UGA) codons in frame. We subsequently filtered these putative coding regions using the same criteria as for RefSeq annotated sequences as described above. For *Chlamydomonas*, such de novo construction of coding sequences finds 97.5% of the known coding sequences that pass the same filtering criteria (50 reads mapping, at least 10 unique hits) and of these, 96.7% are exact matches to those in RefSeq. These hits correspond to 25.5% of the total coding sequences recorded for *Chlamydomonas* in RefSeq, but note that not all sequences are being translated in our single, wild-type sample. For mouse, the equivalent figures are 98.1%, 99.1%, and 46.9%, respectively.

### Visualization of uORFs and overlapping ORFs using riboSeqR

**FIGURE 6.**
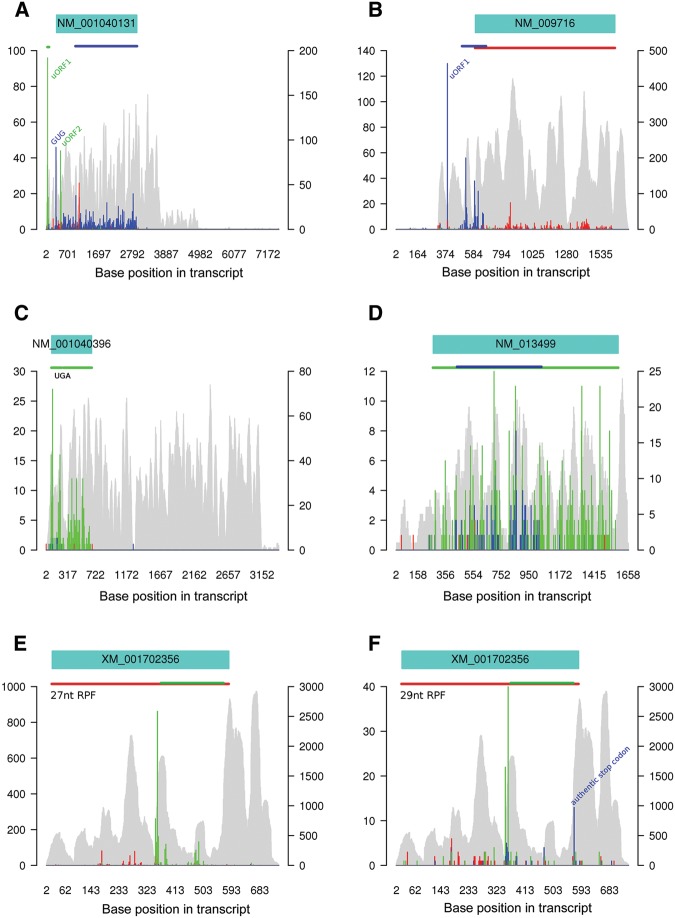
Translated uORFs and overlapping ORFs viewed using riboSeqR. (*A*) Analysis of RPFs mapping to NCBI RefSeq mRNA NM_001040131 (mouse eIF4G2), showing examples of uORFs. Histograms of the 5′ ends of RPFs (colored) and RNA-seq (gray) reads are shown. The three reading frames to which the 5′ ends of RPFs may map to (relative to nt 1 of the reference sequence) are indicated by different colors, as in Figure 4B. The positions of putative ORFs with at least 50 RPFs mapping to at least 10 locations are shown immediately *above* the histograms, color-coded as appropriate. A 15-codon sliding window mean of filtered (see Materials and Methods) Ribo-seq CHX RPF counts is shown *below* the transcript map. The main coding ORF for this transcript is in frame 2 (blue), but translation initiates upstream of the annotated ORF (at the indicated GUG codon). Two uORFs are apparent in frame 1 (green) and an overlapping ORF is also observed (red, see text). (*B*) Analysis of NM_009716 (mouse ATF4) reveals the two regulatory uORFs (see text), uORF1 and uORF2 (blue), upstream of the main coding sequence (red). (*C*) Analysis of NM_001040396 (mouse Sel T) showing an example of translation beyond a canonical stop codon by selenocysteine insertion at an in-frame UGA codon (indicated). Note this gene has proportionately a very long 3′ UTR. (*D*) An example of a likely internal overlapping gene from NM_013499 (Cr1l). (*E*,*F*) riboSeqR analysis of Rubisco expression as an example demonstrating a sequencing error in the RefSeq. This error results in an apparent change of reading frame of the 27-nt RPF ribosome profile. The lack of frame 1 (red) reads within the internal ORF and the clear detection of the de facto termination peak at position 568 (using the 29-nt data; *F*) confirms the RefSeq error.

Ribosome profiling permits the identification of short, translated ORFs upstream of “main” coding sequence (uORFs), some of which utilize near-cognate, non-AUG initiation codons (e.g., CUG) (Ingolia et al. 2011). It can also facilitate the discovery of short ORFs that lack a specific initiation codon but are instead accessed via noncanonical translation mechanisms (Michel et al. 2012; Gerashchenko et al. 2012). RPFs immediately after a canonical stop codon may derive from stop codon read-through (Dunn et al. 2013) or ribosomal frameshifting at or near the stop codon. Figure 6 shows a number of validated examples of such translation events. In each case, riboSeqR was used to identify and display the relevant reads from our data files following input of the chosen accession number. In each panel, RNA-seq reads for a particular gene are shown in gray with Ribo-seq reads superimposed in three colors, representing RPFs whose 5′ ends map to each of the three possible codon positions. Above each panel, the annotated NCBI Reference Sequence Database (RefSeq) ORF is displayed as a turquoise box. Colored lines above the panels show the coding sequences inferred with riboSeqR. We then identified cases in which the filtered putative coding sequence did not correspond to the annotated coding sequence. These cases may represent either misannotation of the transcriptome or transcriptomic sequence or more interestingly, alternative transcription/translation events.

Figure 6A shows mouse initiation factor eIF4G2, the translation of which is initiated from a GUG codon (Takahashi et al. 2005). This is clearly evident in the plot, with abundant reads at the GUG followed by a continuum of reads in this frame up to the stop codon. We also noticed two other highly utilized AUG codons in the 5′ leader of eIF4G2 that would initiate translation of short uORFs (with 16 and seven codons, respectively) that could be regulatory. Indeed, the major peak in the eIF4G2 plot mapped to the most 5′ uORF (in green). The noticeable spike in red, however, does not correspond to a start codon (canonical or otherwise), but instead represents a strong termination peak from an overlapping ORF with generally low read counts. Another example of uORF identification is shown in Figure 6B. The 5′ leader of mouse ATF4 (Harding et al. 2000) harbors two uORFs, one very short (three codons) and a longer second (59 codons) uORF overlapping the main ATF4 coding sequence. As can be seen, the majority of reads mapped to the short uORF, consistent with its important regulatory role (Vattem and Wek 2004; Ait Ghezala et al. 2012), and translation of uORF2 and ATF4 was also detectable. Unfortunately, we could not illustrate ribosomal frameshifting or stop codon read-through (see Brierley et al. 2010; Dunn et al. 2013) because read coverage of the relevant mRNAs was too low. However, the transcript for mouse selenoprotein T (SelT) illustrates a specialized form of stop codon read-through (Heider et al. 1992; Kryukov et al. 2003). The efficiency of selenocysteine insertion is very high (Heider et al. 1992; Berry et al. 1993) and, consistent with this, there were similar levels of ribosome footprints in the coding sequences flanking the in-frame stop codon (Fig. 6C). With high levels of framing, the riboSeqR package also allows visualization of likely internal overlapping genes, for example, within the mouse complement component (3b/4b) receptor 1-like protein (Cr1l, also known as mCRY; Paul et al. 1989) (Fig. 6D).

The *Chlamydomonas* genome is less well-annotated than that of the mouse, and riboSeqR allowed us to identify and correct misannotations in RefSeq genes. In our initial analysis, Rubisco small subunit 2 was interpreted as having an internal overlapping gene (Fig. 6E, in green). Indeed, the RefSeq sequence, XM_001702356.1, contains a lengthy internal overlapping ORF. The position of this internal ORF coincided with a change in the phasing of the triplet periodicity of mapped 27-nt RPFs, indicating that translation is predominantly of the internal ORF in the region where it overlaps the annotated Rubisco ORF. However, there is an almost complete absence of RPFs mapping in phase with the main ORF in this region (absence of red spikes, presence of green spikes in the internal ORF of Fig. 6E). Subsequent investigation revealed that 8 nt are missing in the RefSeq, resulting in an incorrectly annotated amino acid sequence from amino acid position 110 onward. A peak in the 29-nt RPF plot (Fig. 6F) at the stop codon of the overlapping ORF in the +2 reading frame supports the idea that this stop codon, and not the annotated stop codon, is the major site of translation termination on the transcript. Although not a new discovery (the corrected amino acid is in agreement with the Uniprot sequence, P08475.1, as well as the latest *Chlamydomonas* transcriptome assembly Cre02.g120150), this example further illustrates the utility of riboSeqR.

## DISCUSSION

Ribosomal RNA contamination of Ribo-seq libraries derives largely from RNase 1 cleavage of surface-exposed regions of the ribosome, which can generate rRNA fragments similar in size to RPFs and leads to their subsequent acquisition during gel size selection. Rigorous RNase 1 digestion to improve triplet periodicity further increases the likelihood of rRNA contamination. The level of contamination varies considerably between different species and experimental protocols. In *Saccharomyces cerevisiae*, ∼90% of the reads derive from one fragment of 28S rRNA, and a single round of hybridization-subtraction using an antisense oligonucleotide targeting this sequence is sufficient to remove much of it (Gerashchenko et al. 2012). In other organisms, however, such as human and mouse embryonic stem cells, the contaminants are more complex and libraries substantially enriched in specific RPFs can only be obtained if 10–20 rRNA fragments are subtracted by hybridization (Ingolia et al. 2012, 2013). Recently, commercial rRNA depletion kits (i.e., RiboZero) have also been used to deplete Ribo-seq libraries with some success (Bazzini et al. 2014; Smith et al. 2014). The use of DSN outlined in the present study offers an alternative approach to enrich for mRNA-specific RPFs in profiling libraries.

In a side-by-side comparison, we found DSN was able to deplete rRNA to a level comparable to RiboZero treatment (nine- to 11-fold versus 18-fold enrichment of mRNA, respectively; Fig. 3A). DSN was found to bias the most highly abundant individual mRNA read species (Fig. 3D), but, on the other hand, RiboZero treatment was found to bias individual mRNA read species that showed complementarity to the RiboZero probes (Fig. 3B). On a whole-transcript level, neither DSN nor RiboZero led to appreciable bias in the mRNA population (Fig. 3C), presumably due to biased individual RPF species being a relatively small fraction of the total number of RPF species even in the most highly expressed mRNAs. DSN treatment may be particularly useful for non-model organisms as it obviates the necessity to identify major rRNA contaminants in advance for hybridization-subtraction approaches. Furthermore, it will also be useful in situations where the rRNA contamination is diverse. With *C. reinhardtii*, for example, the complexity of contaminating sequences (derived mostly from the highly abundant cytoplasmic ribosomes, but including mitochondrial and chloroplastic rRNA sequences) rendered hybridization-subtraction methods inadequate (data not shown). DSN could also deplete other highly abundant contaminants that may be present in some samples, such as fragments of tRNAs, U2 snRNA, or snoRNAs. Furthermore, DSN is simple to use and can also be used in conjunction with other enrichment methods.

The riboSeqR R package was developed to enable non-specialists to parse aligned Ribo-seq data, to identify the predominant lengths of ribosomal fragments and the codon positions to which they map, and to thus identify coding sequences undergoing translation. A plethora of visualization methods are provided to facilitate this task and summarize the behavior of the ribosome, both on average (i.e., summed over many mRNAs; Figs. 4, 5) and for individual transcripts (Fig. 6). We used the mouse and *Chlamydomonas* data sets here to show the versatility of the package in the identification and visualization of established examples of noncanonical translation. Good triplet periodicity in Ribo-seq data sets is advantageous in the identification by riboSeqR of previously unannotated ORFs (Gerashchenko et al. 2012; Michel et al. 2014) and of overlapping ORFs. The quality of framing is influenced by the extent of RNase 1 digestion and for *Chlamydomonas*, over a range of RNase 1 concentrations between 600 and 1600 units per mL of lysate (*A*_254_ = 4), we found that the most abundant RPFs showed framing between 85% and 96% (data not shown). The digestion conditions utilized for the mouse lysates were identical to those described by Ingolia et al. (2012) and generated good framing without further optimization.

Identification and assessment of differential translation, in which the ratio of translation as assessed by Ribo-seq to transcription as assessed by RNA-seq varies between biological conditions, present the next challenges in understanding translation regulation. In this paper, we have considered data from a single biological sample and so differential expression analyses are not relevant. However, methods are provided in the riboSeqR package for extracting count data for translated coding sequences for analysis of differential translation by summing over specific size class/frame combinations. These data may be paired with counts from RNA-seq data from the same samples for analyses of differential translation efficiency, which we suggest may be achieved through methods for analysis of paired high-throughput sequencing data, as implemented in the BaySeq R package (Hardcastle and Kelly 2013).

## MATERIALS AND METHODS

### Ribosomal profiling

The profiling methodologies used were based largely on those described by Ingolia and colleagues (Ingolia et al. 2009, 2012), except library amplicons were constructed using a small RNA cloning strategy (Guo et al. 2010) adapted to Illumina smallRNA v2 to allow multiplexing.

### Cell culture and lysis

*Chlamydomonas reinhardtii* cells (CC-4350 cw[15] Arg 7–8 mt^+^) (Chlamydomonas Resource Center: http://chlamycollection.org/strains/) were maintained in 750 mL Tris–acetate–phosphate medium (Harris 1989) at 23°C on a rotatory shaker (140 rpm) under constant illumination with white light (70 µE m^2^ sec^−1^) to mid-log phase (OD_750_∼0.6). Cultures were harvested by filtering off the media, the cell paste was flash frozen and pulverized in liquid nitrogen with 5 mL of prefrozen lysis buffer (20 mM Tris–Cl pH7.5, 140 mM KCl, 5 mM MgCl_2_, 100 µg/mL cycloheximide, 100 µg/mL chloramphenicol, 0.05 mM DTT, 0.1% NP40 and 5% sucrose). The frozen powder was thawed on ice and clarified by centrifugation for 30 min at 4700 rpm at 4°C followed by adjustment of *A*_254_ to ∼4 before snap-freezing in liquid nitrogen and storage at −80°C.

Murine 17 clone 1 cells were maintained in Dulbecco's modification of Eagle's medium supplemented with 10% (vol/vol) fetal calf serum. Cells (10^7^) were plated in a 10 cm dish and upon reaching 100% confluence, cycloheximide (Sigma-Aldrich) was added to 100 µg/mL. After 2 min, cells were rinsed with 5 mL of ice-cold PBS, the dishes submerged in a reservoir of liquid nitrogen for 10 sec, transferred to dry ice and 400 µL of lysis buffer (20 mM Tris–HCl pH 7.5, 150 mM NaCl, 5 mM MgCl_2_, 1 mM DTT, 1% Triton X-100, 100 µg/mL cycloheximide and 25 units/mL TURBO DNase [Life Technologies]) dripped on. The cells were scraped extensively to ensure lysis, collected and triturated with a 26-G needle 10 times. Lysates were clarified by centrifugation for 20 min at 13,000*g* at 4°C, the supernatants recovered and stored in liquid nitrogen.

### Nuclease footprinting

For *Chlamydomonas*, lysates were slowly thawed on ice and a 200 µL aliquot (*A*_254_ = 4) treated with 300 units RNase 1 (100 units/µL, Life Technologies cat. no. AM2294) in a thermo-mixer at 28°C, 400 rpm for 30 min. The tube was placed on ice, 2 µL of SUPERase-In RNase inhibitor (20 units/mL, Life Technologies) added, and the reaction was layered onto a 1 M sucrose cushion prepared in *Chlamydomonas* polysome buffer (20 mM Tris–HCl pH 7.5, 140 mM KCl, 5 mM MgCl_2_, 0.5 mM DTT, 100 µg/mL cycloheximide, 100 µg/mL chloramphenicol, and 0.5 µg/mL SUPERase-In). The cushion was ultracentrifuged at 38,000 rpm (5 h, 4°C) in a Beckman Sw41Ti rotor. For mouse samples, lysates were slowly thawed on ice and 300 µL treated with 7.5 µL RNase 1 followed by incubation for 45 min at room temperature on a rotating wheel. Ten microliters of SUPERase-In RNase inhibitor was added, the sample was layered onto a 1 M sucrose cushion in mammalian polysome buffer (20 mM Tris–HCl pH 7.5, 150 mM NaCl, 5 mM MgCl_2_, 1 mM DTT, 100 µg/mL cycloheximide) and ultracentrifuged at 28,000 rpm (16 h, 4°C) in a Beckman SW55Ti rotor. Subsequently, all ribosome pellets were resuspended in 200 µL of the corresponding polysome buffer and digested with proteinase K (10 mM Tris–HCl pH 7.5, 10% SDS, 200 µg/mL Proteinase K [New England BioLabs]) for 30 min at 42°C. RPFs were recovered by extracting twice with prewarmed (65°C) acidic phenol:chloroform (Life Technologies) and once with chloroform (1:1, vol/vol, buffered with 10 mM Tris pH 7.5, 0.1 mM EDTA) followed by ethanol precipitation. RPFs were resuspended in 10 mM Tris–HCl pH 7.5 and quantified by spectrophotometry.

### Purification of total RNA and fragmentation by alkaline hydrolysis

Two hundred microliters of each cell lysate was digested with proteinase K and cellular RNA extracted using acidic phenol:chloroform as above. Ribosomal RNA was depleted from 5 µg of total RNA using a RiboZero rRNA removal kit targeting the appropriate species following the manufacturer's instructions (Human/Mouse/Rat: Epicentre, cat. no. RZH1046, Plant Seed/root: Epicentre, cat. no. MRZSR116). Depleted RNA was resuspended in 10 mM Tris–HCl pH 7.5 and quantified by spectrophotometry. A measure of 1–2 µg of total RNA in 20 µL was mixed with an equal volume of 2× alkaline fragmentation solution (2 mM EDTA, 10 mM Na_2_CO_3_, and 90 mM NaHCO_3_) and incubated for 15 min at 95°C. The reaction was diluted by addition of 280 µL stop/precipitation solution (300 mM NaOAc pH 5.5, GlycoBlue Co-precipitant [Ambion, 15 mg/mL]), and fragmented RNA recovered by ethanol precipitation.

### RNA size selection

Fragmented total RNA or RPFs (1–2 µg) were separated on 15% (wt/vol) denaturing polyacrylamide gels and RNA species migrating between 28 and 34 nt were harvested. RNA was eluted from the gel slices on a rotator overnight at 4°C, in 600 µL RNA gel extraction buffer (300 mM NaOAc pH 5.5, 1 mM EDTA, and 0.25% SDS). Eluted RNA was ethanol precipitated as described above.

### Generation of RNA libraries

The RNA samples from above were heated at 80°C for 2 min, cooled and the 3′ phosphate group removed using T4 polynucleotide kinase (T4 PNK, New England BioLabs) for 2 h at 37°C in a 20 µL reaction lacking ATP. The RNA was concentrated by ethanol precipitation, resuspended in 10 mM Tris–HCl pH 7.5 and ligated in a 20 µL reaction overnight at 14°C to a preadenylated 3′-adaptor (5′-rATGGAATTCTCGGGTGCCAAGG-3′) using T4 RNA Ligase 2 truncated K227Q (New England BioLabs). This 3′ adaptor was adenylated using a 5′ DNA adenylation kit (New England BioLabs) following the manufacturer's instructions. RNA was precipitated, loaded into a 15% (wt/vol) denaturing polyacrylamide gel and ligated RNA fragments migrating between 49 and 53 nt excised. The RNA was eluted, precipitated, resuspended as above, and 5′ phosphorylated using T4 PNK in the presence of 1 mM ATP for 2 h at 37°C. RNA was concentrated by ethanol precipitation, resuspended in 10 mM Tris–HCl pH 7.5 and ligated to a 5′ RNA adaptor (5′-rGrUrUrCrArGrArGrUrUrCrUrArCrArGrUrCrCrGrArCrGr ArUrC-3′) in a 20 µL reaction overnight at 14°C using T4 RNA Ligase (Promega). The fully adapted RNAs were recovered by ethanol precipitation, dissolved in 6 µL 10 mM Tris–HCl pH 7.5, and 3 µL of this ligated product annealed to an RT primer (5′-GCCTTGGCACCCGAGAATTCCA, 50 pmol) for 5 min at 65°C. The RNA was subsequently reverse transcribed for 50 min at 55°C in a 20 µL reaction following addition of first strand buffer (Invitrogen, to 1×), 2.5 mM MgCl_2_, 10 mM DTT, 0.5 µL SUPERase-In, and 1 µL SuperScript III (Invitrogen), followed by heat inactivation for 5 min at 85°C.

### PCR amplification and barcode addition

Standard PCR reactions were used to prepare amplicons using forward primer RP1 (5′-AATGATACGGCGACCACCGAGATCTACA CGTTCAGAGTTCTACAGTCCGA-3′) and 5′-CAAGCAGAAGAC GGCATACGAGATN_6_GTGACTGGAGTTCCTTGGCACCGAGAA TTCCA-3′ (RPIX) as reverse primer, where X is primer number (1–24) and N_6_ the reverse complement of the respective hexanucleotide index sequence detected during Illumina sequencing. *Chlamydomonas* PCR amplification used New England BioLabs (NEB) Q5 2X master mix (because of high GC content) and was performed using a ramp-rate of 2.2°C/sec with the following cycling conditions: one cycle of 98°C for 3 min, 13 cycles of 98°C for 1 min, 65°C for 30 sec, and 72°C for 30 sec, followed by an elongation step of 72°C for 5 min. PCR of mouse cDNA used Phusion High-Fidelity PCR Master Mix (NEB) and comprised one cycle of 98°C for 30 sec, 13 cycles of 98°C for 10 sec, 60°C for 30 sec, and 72°C for 15 sec, followed by an elongation step of 72°C for 10 min. PCR reactions were loaded onto 10% nondenaturing polyacrylamide-TBE gels and run for 45 min at 12 W. Products of ∼150 bp were excised from the gel and eluted at 4°C overnight on a rotator following addition of 600 µL DNA gel extraction buffer (300 mM NaCl, 10 mM Tris–HCl pH 8 and 1 mM EDTA). These amplicon libraries were ethanol precipitated and resuspended in 15 µL 10 mM Tris–HCl pH 7.5. Libraries were sequenced using Illumina HiSeq2000, NextSeq500, or MiSeq platforms.

### Ribosomal RNA depletion

#### RiboZero-based rRNA subtraction

Following nuclease footprinting, RPFs (2 µg) were subjected to RiboZero treatment as detailed above for total cellular RNA. Subsequent gel purification of appropriately sized RPFs and library amplicon generation was carried out in parallel with an undepleted library to allow unambiguous band identification.

#### Antisense oligonucleotide (AON)-based rRNA subtraction

Following adaptor-ligation and reverse transcription, major rRNA sequence contaminants in library cDNAs were targeted by annealing to a pool of 14 biotinylated AONs and subsequently removed using streptavidin beads as previously described (Ingolia et al. 2012).

#### Treatment with duplex-specific nuclease (DSN)

Ribosomal RNA was depleted from Ribo-seq samples at the library amplicon stage; 12 µL of the relevant Ribo-seq library was mixed with 4 µL of 4× hybridization buffer (200 mM HEPES pH 7.5 and 2 M NaCl) and denatured at 98°C for 2 min. DNA was re-annealed for 5 h at 68°C prior to addition of 2 µL of 10× DSN master buffer and 2 µL of DSN (4 units, Evrogen). Digestion was allowed to proceed for 25 min at 68°C (mouse) or up to 90 min (*Chlamydomonas*), before addition of 20 µL 10 mM EDTA and incubation for a further 5 min at 68°C. DNA was recovered by a single extraction with phenol–chloroform (1:1, vol/vol) followed by ethanol precipitation and resuspended in 4 µL 10 mM Tris–HCl pH 7.5. The treated amplicon library was subjected to another round of PCR (as above) and the resulting library sequenced or subjected to a second round of DSN treatment.

### Bioinformatic analysis

Adaptor sequences were trimmed using the FastX-toolkit. To remove remaining post-DSN contamination, trimmed reads were first mapped to *C. reinhardtii* and *Mus musculus* (as appropriate) databases of rRNA and common noncoding RNAs (ncRNAs), using Bowtie version 1 (Langmead et al. 2009) with seed length 23. In order to select good-quality samples of nuclear-encoded, mitochondrial and chloroplastic mRNA-derived RPFs, remaining reads were mapped to *C. reinhardtii* and *M. musculus* NCBI RefSeq mRNAs, and organellar coding ORFs derived from NCBI RefSeq *C. reinhardtii* and *M. musculus* organellar genomes. No specific consideration was given to the presence of multiple isoforms within the RefSeq database: Each read that could be mapped to multiple transcripts was assigned at random to one of these transcripts. The Bowtie alignment was used as input for riboSeqR, along with a FASTA file containing the transcriptome of interest, to generate Figures 4–6.

RPF framing distributions produced with riboSeqR (Figs. 4B, 5B) were derived from reads mapping to the “interior” regions of annotated coding ORFs; specifically the entire read had to be contained within the ORF, thus, in general, excluding RPFs of initiating or terminating ribosomes. Histograms of 5′ end positions of RPFs relative to start and stop codons were derived from reads mapping to RefSeq mRNAs with at least 50 reads of the most abundant read-length size class mapping in frame in at least 10 separate locations in the ORF. For a given length size class, the values shown represent a weighted average of the abundance of reads mapping on all selected transcripts.

For the comparison of rRNA depletion strategies (Figs. 2, 3), library composition was determined by mapping reads to rRNA, mRNA, and genomic (gDNA) databases. Remaining reads that mapped to gDNA presumably derived from ncRNAs and unannotated transcripts (both ncRNA and mRNA). Minimum free folding energies of individual reads were calculated using RNAfold from the ViennaRNA package (Hofacker 2003). Since DSN is applied post-adaptor-ligation and post-RT-PCR, the minimum free energy of reads was calculated using DNA energy parameters (dna_mathews2004.par). The untreated and test libraries were sequenced together using unique multiplex tags at nt 34–39 of the 63 nt 3′ adaptor sequence. Since the multiplex tag produces a systematic bias in the calculated MFE that varies from one sample to another, the MFE was calculated for each read in the context of the 5′ adaptor and just the first 33 nt of the 3′ adaptor. Optimal RNA:RNA binding energies between reads and reverse-complemented rRNA (RiboZero) were calculated using RNAduplex from the ViennaRNA package with default RNA energy parameters. For both RNAfold and RNAduplex, the temperature parameter was set to 68°C, the annealing temperature used in the DSN and RiboZero protocols.

## DATA DEPOSITION

The Ribo-seq data have been deposited in the ArrayExpress database (http://www.ebi.ac.uk/arrayexpress) under accession numbers E-MTAB-2934 and E-MTAB-3583.

## SUPPLEMENTAL MATERIAL

Supplemental material is available for this article.

## Supplementary Material

Supplemental Material
