# Supplementary material for: The use of duplex-specific nuclease in ribosome profiling and a user-friendly software package for Ribo-seq data analysis
Source: RNA. 2015 Oct;21(10):1731–45. doi: 10.1261/rna.052548.115 (PMC4574750; doi:10.1261/rna.052548.115)
Supplement: Supplemental Material [file supp_21_10_1731__index.html]

The use of duplex-specific nuclease in ribosome profiling and a user-friendly software package for Ribo-seq data analysis — The use of duplex-specific nuclease in ribosome profiling and a user-friendly software package for Ribo-seq data analysis — Supplemental Material 

# The use of duplex-specific nuclease in ribosome profiling and a user-friendly software package for Ribo-seq data analysis

## Supplemental Material

**Files in this Data Supplement:**

- Supp Fig 1.pdf
- Supp Fig 2.pdf
