# Supplementary material for: The use of duplex-specific nuclease in ribosome profiling and a user-friendly software package for Ribo-seq data analysis
Source: RNA. 2015 Oct;21(10):1731–45. doi: 10.1261/rna.052548.115 (PMC4574750; doi:10.1261/rna.052548.115)
Supplement: Supplemental Material [file supp_052548.115_Supp_Fig_1.pdf]

riboSeqR: analysis of chlamydomonas data

Load riboSeqR library.

```
library(riboSeqR)
```

Specify bowtie output files aligning ribosomal and RNA-seq data to the transcriptome.

```
ribofile <- "RiboSeq-WT-2/mRNA.bowtie"
rnafile <- "RNASeq-WT-2/mRNA.bowtie"
```

Read the data.

```
riboDat <- readRibodata(ribofile, rnafile, replicates = "wt1",
    columns = c(strand = 2, seqname = 3, start = 4,
        sequence = 5))

## Reading ribosomal files....done!
## Reading rna files....done!
```

Plot the distribution of lengths in the ribosomal data.

```
lengthDist(riboDat, lwd = 3, cex = 1.5, cex.lab = 1.5,
    cex.axis = 1.5, ylim = c(0, 0.6))
```

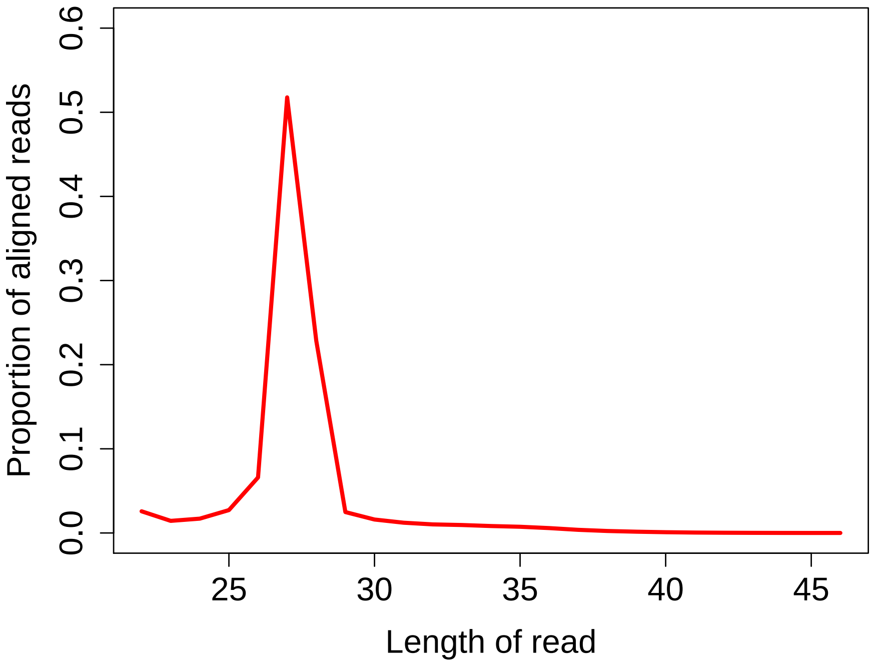

Read in pre-specified (RefSeq) CDS coordinates for each transcript, and specify frame.

```
CDS <- read.delim(file = "chlamy_CDS.txt", as.is = TRUE)
CDS <- GRanges(CDS$seqnames, IRanges(start = CDS$start,
    end = CDS$end))
CDS$frame <- (start(CDS) - 1)%%3
```

Count ribosomal hits within each coding sequence.

```
fCs <- frameCounting(riboDat, CDS, lengths = 16:42)

## Calling frames..........done!
```

Examine abundances of ribosomal hits within coding sequence, split by read length and frame.

```
fS <- readingFrame(rC = fCs, lengths = 22:37)
```

Filter on sufficient number of hits of maximum length (27mer) in the correct frame (1).

```
ffCs <- filterHits(fCs, fS = fS, lengths = 27, frames = 1,
    hitMean = 50, unqhitMean = 10)
```

Plot abundances of ribosomal hits within coding sequences and frames.

```r
plotFS(fS, main = "nuclear", space = c(0, 0.5), cex.names = 1.5,
    args.legend = list(cex = 1.5, bty = "n", border = rainbow(3,
        s = 0.7)))
```

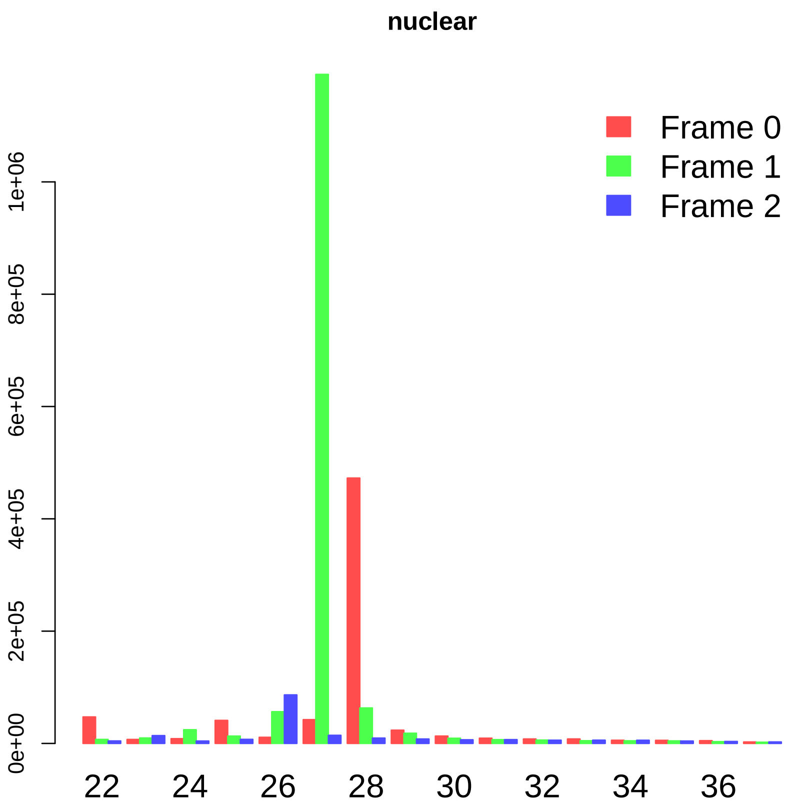

Plot average distribution of read starts over filtered transcripts at 5' and 3' ends.

```r
par(mfrow = c(3, 1))
plotCDS(coordinates = ffCs@CDS, riboDat = riboDat,
    lengths = c(27), max5p = 400, main = "27nt", min5p = -100)
plotCDS(coordinates = ffCs@CDS, riboDat = riboDat,
    lengths = c(28), max5p = 400, main = "28nt", min5p = -100)
plotCDS(coordinates = ffCs@CDS, riboDat = riboDat,
    lengths = c(29), max5p = 400, main = "29nt", min5p = -100)
```

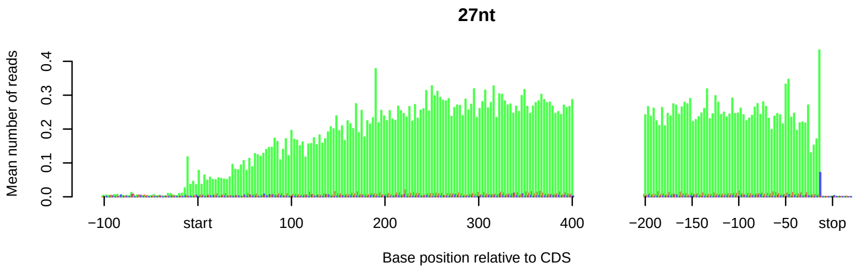

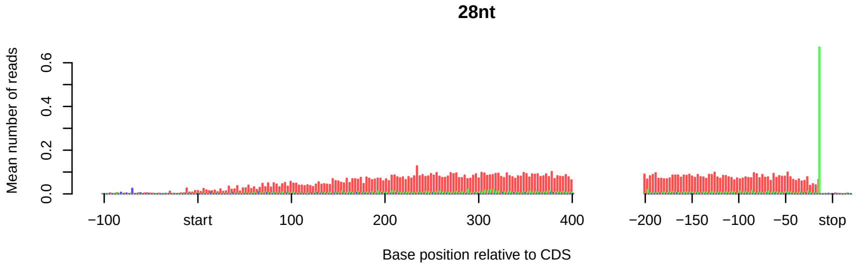

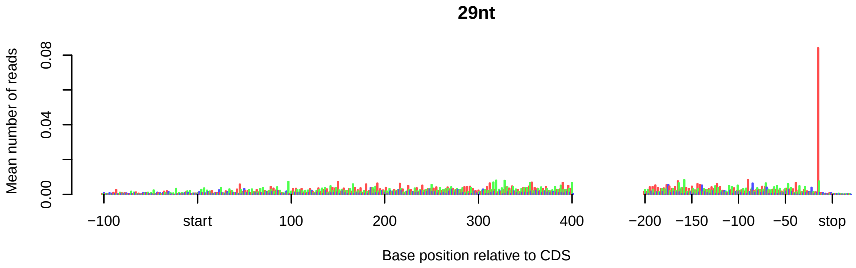

Construct *de novo* putative CDS coordinates by inspecting transcriptome for canonical start/stop codons (in the same frame).

```
novoCDS <- findCDS(fastaFile = "Chlamydomonas_reinhardtii/mRNA/REFSEQ/mRNA.fasta",
    startCodon = c("ATG"), stopCodon = c("TAG", "TAA",
        "TGA"))
```

Assign ribosomal reads to putative CDS coordinates and filter.

```
fCs.novo <- frameCounting(riboDat, novoCDS, lengths = 16:42)

## Calling frames..........done!

ffCs.novo <- filterHits(fCs.novo, lengths = 27, frames = 1,
    hitMean = 50, unqhitMean = 10, fS = fS)
```

Examine the specificity and sensitivity of the de novo identification of coding sequences, by comparison to those present in the (filtered) annoted coding sequences. We can also compare to the total population of known coding sequences; this will inevitably be considerably larger as not all coding sequences will be translated in this data.

```r
existing <- sum(ffCs.novo@CDS %in% ffCs@CDS)
existing / length(ffCs.novo@CDS) * 100

## [1] 96.65012

existing / length(ffCs@CDS) * 100

## [1] 97.49687

existing / length(fCs@CDS) * 100

## [1] 25.47972
```

Examine those novel putative coding sequences where more than one coding sequence is predicted for a given transcript.

```r
gene.novo <- sub("(_[0-9]*?\\.\\.).*", "", as.character(seqnames(ffCs.novo@CDS)))
dupgen <- which(gene.novo %in% gene.novo[duplicated(gene.novo)])
dupgen <- dupgen[order(gene.novo[dupgen])]

par(mfrow = c(4, 1))
for (tran in unique(as.character(seqnames(ffCs.novo@CDS))[dupgen])[1:4]) {
    breakdown <- strsplit(tran, "_|\\.\\.")[[1]]
    rectCDS <- GRanges(seqnames = paste(breakdown[1:2],
        collapse = "_"), IRanges(start = as.numeric(breakdown[3]),
        end = as.numeric(breakdown[4])))
    bw <- plotTranscript(tran, coordinates = ffCs.novo@CDS,
        annotation = rectCDS, riboData = riboDat, length = 27,
        frameShift = 1, baseLim = c(1, as.numeric(breakdown[[5]])),
        main = "")
}
```

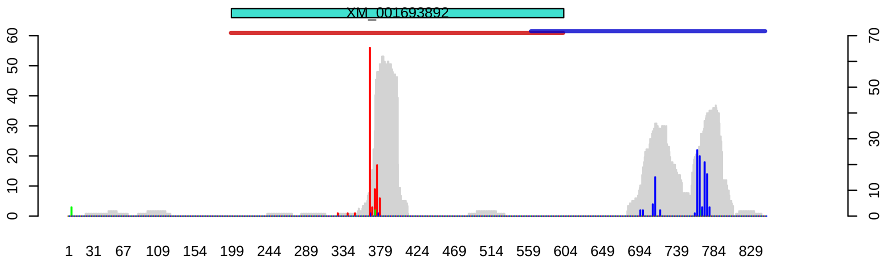
XM_001693892

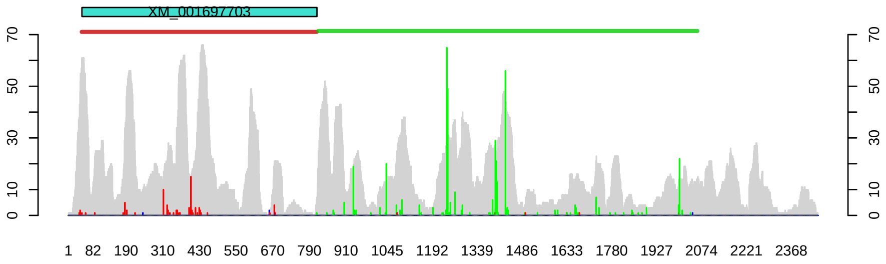
XM_001697703

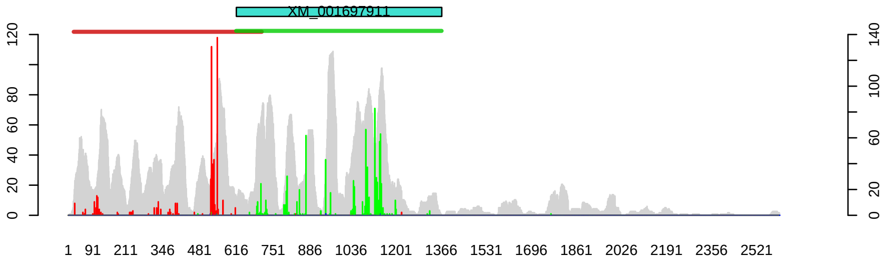
XM_001697911

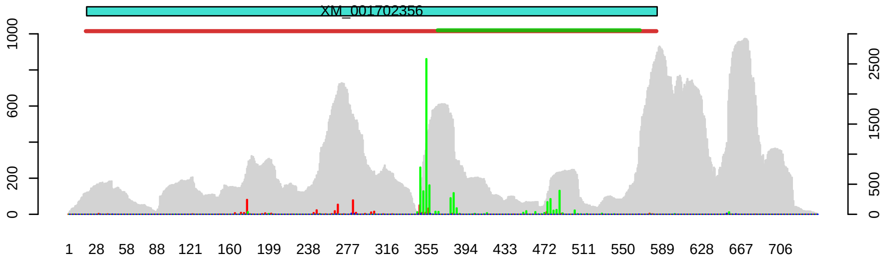
XM_001702356

Total processing time for these data:

```
## Time difference of 14.04966 mins
```

Session info:

```
## R version 3.1.0 (2014-04-10)
## Platform: x86_64-unknown-linux-gnu (64-bit)
##
## locale:
##  [1] LC_CTYPE=en_GB.UTF-8
##  [2] LC_NUMERIC=C
##  [3] LC_TIME=en_GB.UTF-8
##  [4] LC_COLLATE=en_GB.UTF-8
##  [5] LC_MONETARY=en_GB.UTF-8
##  [6] LC_MESSAGES=en_GB.UTF-8
##  [7] LC_PAPER=en_GB.UTF-8
##  [8] LC_NAME=C
##  [9] LC_ADDRESS=C
## [10] LC_TELEPHONE=C
## [11] LC_MEASUREMENT=en_GB.UTF-8
## [12] LC_IDENTIFICATION=C
##
## attached base packages:
## [1] stats4    parallel  stats     graphics
## [5] grDevices utils     datasets  methods
## [9] base
##
## other attached packages:
## [1] riboSeqR_1.0.0        abind_1.4-0
## [3] GenomicRanges_1.17.48 GenomeInfoDb_1.1.26
## [5] IRanges_1.99.32       S4Vectors_0.2.8
## [7] BiocGenerics_0.11.5   knitr_1.7
##
## loaded via a namespace (and not attached):
## [1] evaluate_0.5.5 formatR_1.0    highr_0.3
## [4] stringr_0.6.2  tools_3.1.0    XVector_0.5.8
```
