# Supplementary material for: The use of duplex-specific nuclease in ribosome profiling and a user-friendly software package for Ribo-seq data analysis
Source: RNA. 2015 Oct;21(10):1731–45. doi: 10.1261/rna.052548.115 (PMC4574750; doi:10.1261/rna.052548.115)
Supplement: Supplemental Material [file supp_052548.115_Supp_Fig_2.pdf]

riboSeqR: analysis of mouse data

Load riboSeqR library.

```
library(riboSeqR)
```

Specify bowtie output files aligning ribosomal and RNA-seq data to the transcriptome.

```
ribofile <- "WTCHG_74295_02_1/mRNA.bowtie"
rnafile <- "WTCHG_74295_04_1/mRNA.bowtie"
```

Read the data.

```
riboDat <- readRibodata(ribofile, rnafile, replicates = "wt1",
    columns = c(strand = 2, seqname = 3, start = 4,
        sequence = 5))

## Reading ribosomal files....done!
## Reading rna files....done!
```

Plot the distribution of lengths in the ribosomal data.

```
lengthDist(riboDat, lwd = 3, cex = 1.5, cex.lab = 1.5,
    cex.axis = 1.5, ylim = c(0, 0.6))
```

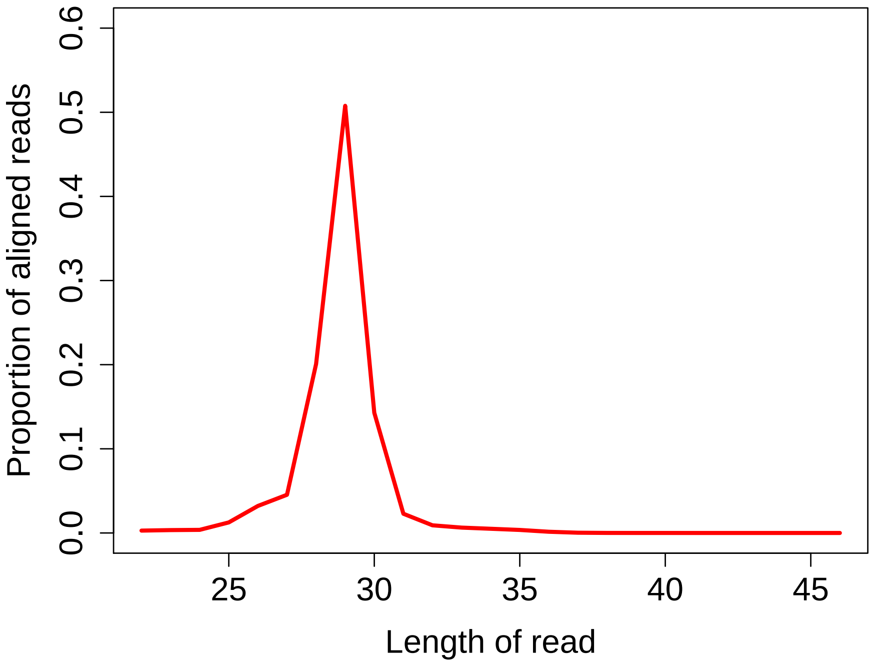

Read in pre-specified CDS coordinates for each transcript, and specify frame.

```
CDS <- read.delim(file = "mouse_CDS.txt", as.is = TRUE)
CDS <- GRanges(CDS$seqnames, IRanges(start = CDS$start,
    end = CDS$end))
CDS$frame <- (start(CDS) - 1)%%3
```

Count ribosomal hits within each coding sequence.

```
fCs <- frameCounting(riboDat, CDS, lengths = 16:42)

## Calling frames..........done!
```

Examine abundances of ribosomal hits within coding sequence, split by read length and frame.

```
fS <- readingFrame(rC = fCs, lengths = 22:37)
```

Filter on sufficient number of hits of maximum length (29mer) in the correct frame (0).

```
ffCs <- filterHits(fCs, fS = fS, lengths = 29, frames = 0,
    hitMean = 50, unqhitMean = 10)
```

Plot abundances of ribosomal hits within coding sequences and frames.

```r
plotFS(fS, main = "nuclear", space = c(0, 0.5), cex.names = 1.5,
    args.legend = list(cex = 1.5, bty = "n", border = rainbow(3,
        s = 0.7)))
```

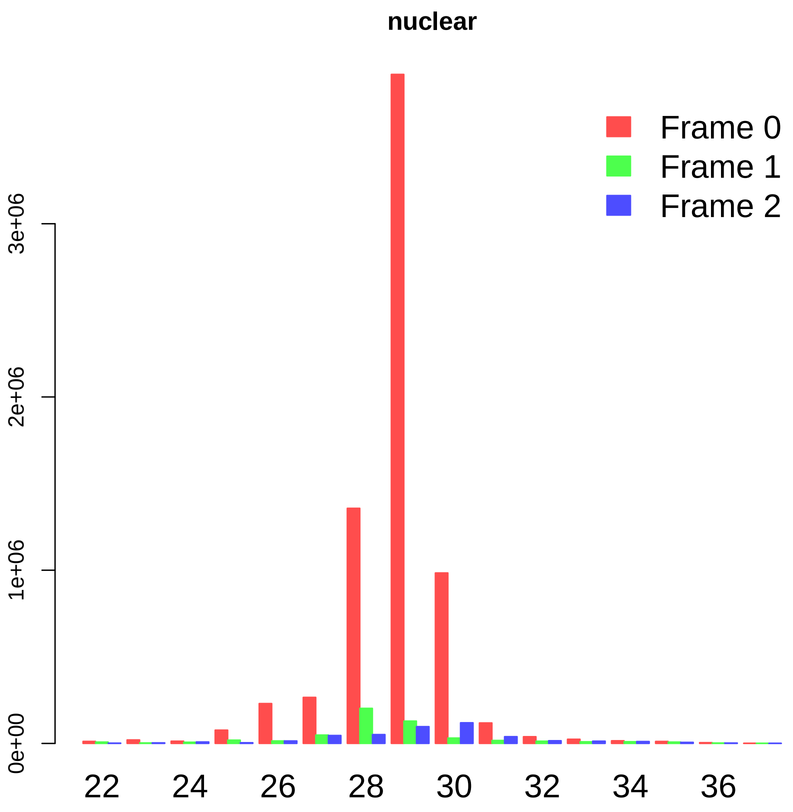

Plot average distribution of read starts over filtered transcripts at 5' and 3' ends.

```r
par(mfrow = c(3, 1))
plotCDS(coordinates = ffCs@CDS, riboDat = riboDat,
    lengths = c(28), max5p = 400, main = "28nt", min5p = -100)
plotCDS(coordinates = ffCs@CDS, riboDat = riboDat,
    lengths = c(29), max5p = 400, main = "29nt", min5p = -100)
plotCDS(coordinates = ffCs@CDS, riboDat = riboDat,
    lengths = c(30), max5p = 400, main = "30nt", min5p = -100)
```

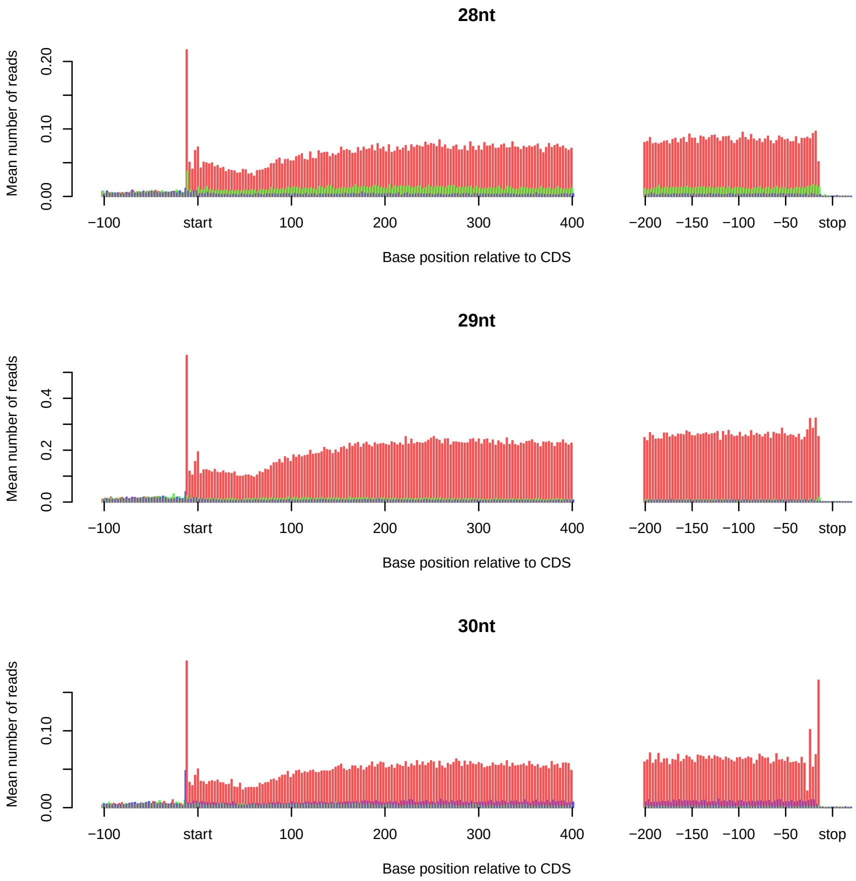

Construct *de novo* putative CDS coordinates by inspecting transcriptome for canonical start/stop codons (in the same frame).

```
novoCDS <- findCDS(fastaFile = "Mus_musculus/mRNA/mRNA.fasta",
    startCodon = c("ATG"), stopCodon = c("TAG", "TAA",
        "TGA"))
```

Assign ribosomal reads to putative CDS coordinates and filter.

```
fCs.novo <- frameCounting(riboDat, novoCDS, lengths = 16:42)

## Calling frames..........done!

ffCs.novo <- filterHits(fCs = fCs.novo, lengths = 29,
    frames = 0, hitMean = 50, unqhitMean = 10, fS = fS)
```

Examine the specificity and sensitivity of the de novo identification of coding sequences, by comparison to those present in the (filtered) annoted coding sequences. We can also compare to the total population of known coding sequences; this will inevitably be considerably larger as not all coding sequences will be translated in this data.

```
existing <- sum(ffCs.novo@CDS %in% ffCs@CDS)
existing/length(ffCs.novo@CDS) * 100

## [1] 98.08719

existing/length(ffCs@CDS) * 100

## [1] 99.0566

existing/length(fCs@CDS) * 100

## [1] 46.87002
```

Examine those novel putative coding sequences where more than one coding sequence is predicted for a given transcript.

```
gene.novo <- sub("(_[0-9]*?\\.\\.).*", "", as.character(seqnames(ffCs.novo@CDS)))
dupgen <- which(gene.novo %in% gene.novo[duplicated(gene.novo)])
dupgen <- dupgen[order(gene.novo[dupgen])]

par(mfrow = c(4, 1))
for (tran in unique(as.character(seqnames(ffCs.novo@CDS))[dupgen])[1:4]) {
    breakdown <- strsplit(tran, "_|\\.\\.")[[1]]
    rectCDS <- GRanges(seqnames = paste(breakdown[1:2],
        collapse = "_"), IRanges(start = as.numeric(breakdown[3]),
        end = as.numeric(breakdown[4])))
    bw <- plotTranscript(tran, coordinates = ffCs.novo@CDS,
        annotation = rectCDS, riboData = riboDat, length = 29,
        frameShift = 0, baseLim = c(1, as.numeric(breakdown[[5]])),
        main = "")
}
```

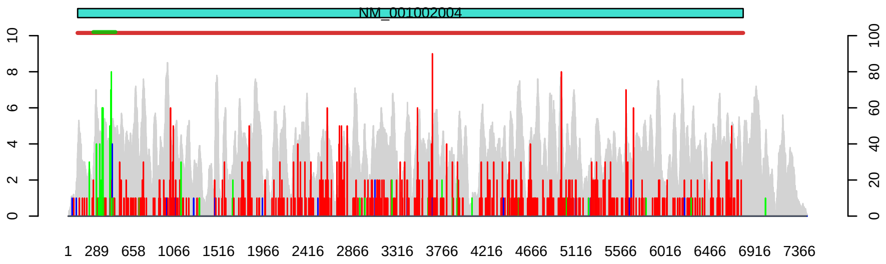
NM_001002004
0 2 4 6 8 10
0 20 40 60 80 100
1 289 658 1066 1516 1966 2416 2866 3316 3766 4216 4666 5116 5566 6016 6466 6916 7366

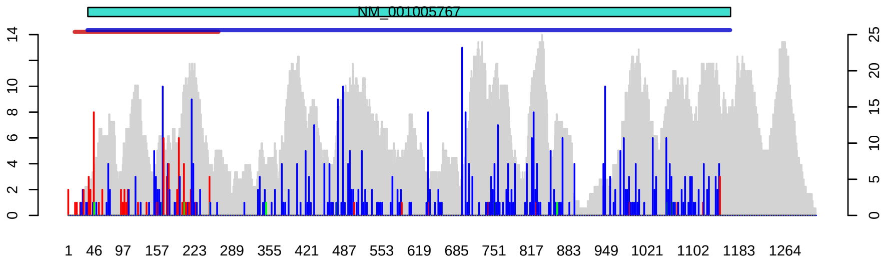
NM_001005767
0 2 4 6 8 10 14
0 5 10 15 20 25
1 46 97 157 223 289 355 421 487 553 619 685 751 817 883 949 1021 1102 1183 1264

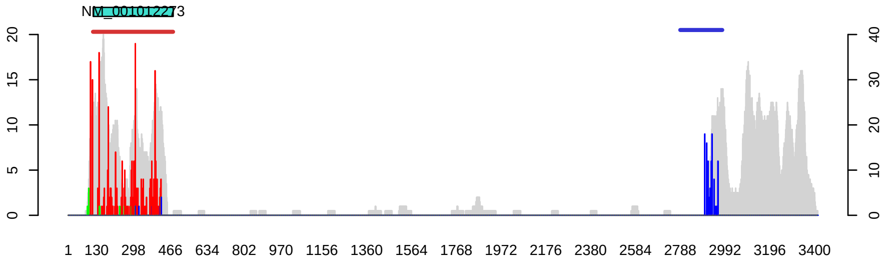
NM_001012273
0 5 10 15 20
0 10 20 30 40
1 130 298 466 634 802 970 1156 1360 1564 1768 1972 2176 2380 2584 2788 2992 3196 3400

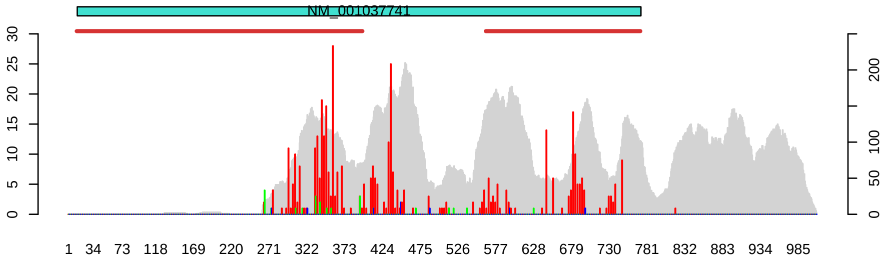
NM_001037741
0 5 10 15 20 25 30
0 50 100 200
1 34 73 118 169 220 271 322 373 424 475 526 577 628 679 730 781 832 883 934 985

Total processing time for these data:

```
## Time difference of 31.07127 mins
```

Session info:

```
## R version 3.1.0 (2014-04-10)
## Platform: x86_64-unknown-linux-gnu (64-bit)
##
## locale:
##  [1] LC_CTYPE=en_GB.UTF-8
##  [2] LC_NUMERIC=C
##  [3] LC_TIME=en_GB.UTF-8
##  [4] LC_COLLATE=en_GB.UTF-8
##  [5] LC_MONETARY=en_GB.UTF-8
##  [6] LC_MESSAGES=en_GB.UTF-8
##  [7] LC_PAPER=en_GB.UTF-8
##  [8] LC_NAME=C
##  [9] LC_ADDRESS=C
## [10] LC_TELEPHONE=C
## [11] LC_MEASUREMENT=en_GB.UTF-8
## [12] LC_IDENTIFICATION=C
##
## attached base packages:
## [1] stats4    parallel  stats     graphics
## [5] grDevices utils     datasets  methods
## [9] base
##
## other attached packages:
## [1] riboSeqR_1.0.0        abind_1.4-0
## [3] GenomicRanges_1.17.48 GenomeInfoDb_1.1.26
## [5] IRanges_1.99.32       S4Vectors_0.2.8
## [7] BiocGenerics_0.11.5   knitr_1.7
##
## loaded via a namespace (and not attached):
## [1] evaluate_0.5.5 formatR_1.0    highr_0.3
## [4] stringr_0.6.2  tools_3.1.0    XVector_0.5.8
```
